# Comprehensive Assessment of Nile Tilapia Skin (*Oreochromis niloticus*) Collagen Hydrogels for Wound Dressings

**DOI:** 10.3390/md18040178

**Published:** 2020-03-25

**Authors:** Baosheng Ge, Haonan Wang, Jie Li, Hengheng Liu, Yonghao Yin, Naili Zhang, Song Qin

**Affiliations:** 1 State Key Laboratory of Heavy Oil Processing and Center for Bioengineering and Biotechnology, China University of Petroleum (East China), Qingdao 266580, China; wang19950410@126.com (H.W.); jieli0532@126.com (J.L.); liu173100@126.com (H.L.); yinyh1996@126.com (Y.Y.); 2 Yantai Institute of Coastal Zone, Chinese Academy of Sciences, Yantai 264003, China; 3 Department of Human Anatomy, School of Basic Medical Science, Binzhou Medical University, 346 Guanhai Road, Laishan, Yantai 264003, China

**Keywords:** collagen hydrogel, fish skin, characterization, wound dressing

## Abstract

Collagen plays an important role in the formation of extracellular matrix (ECM) and development/migration of cells and tissues. Here we report the preparation of collagen and collagen hydrogel from the skin of tilapia and an evaluation of their potential as a wound dressing for the treatment of refractory wounds. The acid-soluble collagen (ASC) and pepsin-soluble collagen (PSC) were extracted and characterized using sodium dodecyl sulphate-polyacrylamide gel electrophoresis (SDS-PAGE), differential scanning calorimetry (DSC), circular dichroism (CD) and Fourier transform infrared spectroscopy (FTIR) analysis. Both ASC and PSC belong to type I collagen and have a complete triple helix structure, but PSC shows lower molecular weight and thermal stability, and has the inherent low antigenicity. Therefore, PSC was selected to prepare biomedical hydrogels using its self-aggregating properties. Rheological characterization showed that the mechanical strength of the hydrogels increased as the PSC content increased. Scanning electron microscope (SEM) analysis indicated that hydrogels could form a regular network structure at a suitable PSC content. Cytotoxicity experiments confirmed that hydrogels with different PSC content showed no significant toxicity to fibroblasts. Skin repair experiments and pathological analysis showed that the collagen hydrogels wound dressing could significantly accelerate the healing of deep second-degree burn wounds and the generation of new skin appendages, which can be used for treatment of various refractory wounds.

## 1. Introduction

Various refractory wounds, such as ulcers and burns, seriously influence the work and life quality of patients, and impose a heavy burden on social security [1,2]. Traditional dry dressing treatments, such as those using absorbent cotton and absorbent gauze, have limited therapeutic effects, and frequent dressing changes can also deepen the suffering of patients. The “wet wound healing theory” proposes that the wet healing environment is beneficial to the growth of the granulation and to facilitating the division of the skin cells, thereby promoting the complete healing of the wound [3,4,5,6,7].

Collagen is the most abundant protein in animals, accounting for about 30% of the total protein [8], which plays a structural role by contributing to the molecular architecture, shape, and mechanical properties of tissues [9]. Because of its low immunogenicity, good biocompatibility and biodegradability [10,11,12], collagen can have a wide range of uses in the field of biomedical materials [13,14,15,16], such as wound dressing for the treatment of different refractory wounds.

When in a neutral solvent, close to physiological temperature, the collagen molecules can spontaneously form collagen fibers, and the hydrogel can be formed by the interaction between the collagen fibers [17,18,19]. Collagen hydrogel has been deemed as a potential wet wound dressing [20]. It possesses the advantages of ordinary hydrogels closely fitting to the wound, preventing bacterial infections, and moisturizing, but also collagen molecules can promote wound epithelialization and accelerate wound healing [21,22,23,24].

Generally, the primary sources of industrial and commercial collagen are limited to land-based mammals, such as pig skin or bovine tendon [25]. However, due to the potential contamination of bovine sponge encephalopathy (BSE), transmissible spongiform encephalopathy (TSE) and foot-and-mouth disease (FMD) [26], coupled with reasons relating to religion [27], many researchers have been exploring alternative sources of collagen that can be derived from marine and freshwater vertebrates or invertebrates [27,28]. Nile tilapia (*Oreochromis niloticus*) is one of the most popularly cultured fish in China, and every year large quantities of collagen-rich fish processing by-products have been discarded [29]. It was reported that the dry weight yields of collagen from tilapia skin could exceed 40% [30,31], which can be used as possible source of collagen.

In recent years, the extraction and application of tilapia collagen has caused extensive studies. Wu et al. [32] have proved that the administration of tilapia type I collagen had low toxicity and confirmed the in vivo biocompatibility for its wide application in biomedical purposes. Yamamoto et al. [33] have proved that all the evaluations of sensitization, cell toxicity, intracutaneous reactions, acute systemic toxicity, pyrogenic reactions, chromosomal aberrations, and hemolysis of the extract of fish (tilapia) collagen gel were negative. Aiah A. et al. [34] prepared the 0.3% and 0.5% (*w*/*v*) Nile tilapia collagen hydrogels and proved that they both showed good biocompatibility with the active proliferation of BHK-21 cells. Zhou et al. [35] had found that biomimetic tilapia collagen nanofibers could promote skin regeneration through inducing keratinocytes differentiation and collagen synthesis of dermal fibroblasts. But there are still many problems that limit the application of collagen hydrogels, especially low mechanical strength making it unsuitable as a block gel [36]. Hou et al. [37,38] have proved that the tilapia collagen showed high biocompatibility and they prepared a composite material using tilapia collagen with konjac glucomannan, which significantly improved its mechanical properties. Although the addition of the second component can significantly improve the mechanical strength of the collagen material, it may decrease the function of collagen. Increasing the collagen content in the hydrogel is another good way to improve mechanical strength, but the hydrogel with extremely high collagen content (> 20 mg/ml) is too dense for cell migration and survival [39]. Therefore, it is still necessary to systematically explore how to prepare collagen hydrogels with good mechanical strength and their healing effect as wound dressing should also be further evaluated.

In this work, the acid-soluble collagen (ASC) and pepsin-soluble collagen (PSC) were extracted from the tilapia skin and then the two collagens were characterized and compared using SDS-PAGE, differential scanning calorimetry (DSC), circular dichroism (CD) and FTIR analysis. A novel hydrogel dressing with high concentration of PSC was prepared using the self-aggregating properties of collagen. The structure and rheological characters of PSC hydrogel were characterized at different PSC contents. The cytotoxicity and skin repair function of collagen hydrogel dressings were also evaluated using cell assay and mouse experiment models. Our experiments provide direct evidence and fundamental data for the application of aquatic origin collagen as wound dressings for treatment of refractory wounds.

## 2. Results and Discussion

### 2.1. Extraction and Characterization of ASC and PSC

**Figure 1:**
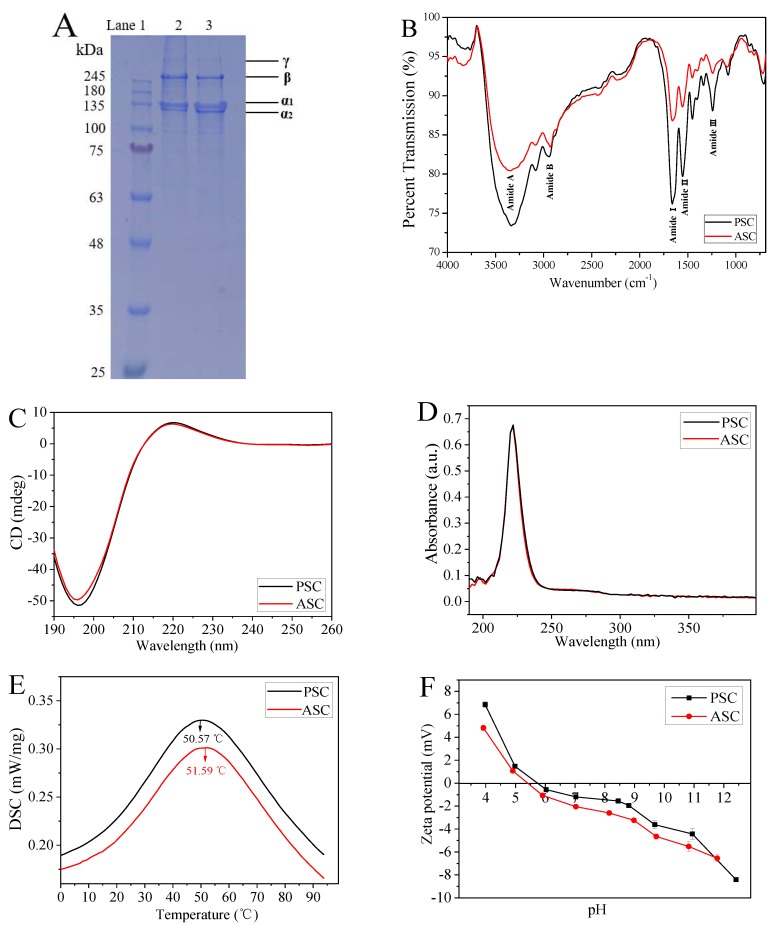
Characterization of ASC and PSC collagens from the skin of tilapia. (**A**): SDS-PAGE analysis of ASC and PSC (Lane 1: protein standards; Lane 2: ASC; Lane 3: PSC). (**B**): FTIR analysis of ASC and PSC. (**C**): Circular dichroism (CD) analysis of ASC and PSC. (**D**): UV–Vis spectra of ASC and PSC. (**E**): Differential scanning calorimetry (DSC) analysis. (**F**): Zeta potential analysis. The isoelectric point (pI) of collagen was determined from the pH value resulting in a zero zeta potential.

The collagen type and complete triple helix structure is the basis for the self-assembly of collagen molecules [17,18], therefore, the extracted ASC and PSC were characterized using SDS-PAGE, FTIR, CD, and UV-Vis experiments. Both ASC and PSC showed four bands on SDS-PAGE, which represent the α_1_, α_2_, β, and γ chains (Figure 1A), and the content of α_1_ was about twice that of α_2_, which was consistent with the molecular composition of type I collagen (α_1_)_2_α_2_ [9,40]. FTIR analysis showed that the major peaks between ASC and PSC were similar (Figure 1B and Table 1), and these peaks corresponded to five major amide linkages, respectively, including amide A, amide B, amide I, amide II, and amide III, which represents the amino acid composition of the collagen molecule and a high proportion of valine and hydroxyproline [29,30]. In addition, the ratio of absorbance derived from the amide III and –CH_2_ bending bands (both are 1452.14 cm^−1^) was about 1.0 (ASC was 1.006 and PSC was 1.012), suggesting the integrity of the intact triple helix structure of collagen [41]. 

It is reported that the ratio of the positive peak to the negative peak (Rpn) in CD spectrum is unique to the structural conformation, which can be used to identify the triple helical structure [42,43,44]. As shown in Figure 1C, the CD spectra of both ASC and PSC exhibited significantly weak positive peaks (220 nm) and strong negative peaks (196 nm), and the Rpn values of ASC and PSC were 0.126 and 0.131, respectively, indicating a triple helical conformation. In Figure 1D, both ASC and PSC have a maximum absorption peak at 222 nm instead of 280 nm due to the low content of tyrosine and phenylalanine [29], which is also deemed as one of the characteristics of collagen.

DSC analysis showed that there was only one endothermal peak within the temperature range of 0–100 °C for both PSC and ASC (Figure 1E), which is related to the thermal denaturation temperature of collagen and the water bonded to collagen molecules [45,46]. The denaturation temperature of PSC was 50.57 °C, which was lower than that of ASC (51.59 °C). Zeta potential values of collagens at various pH values are shown in Figure 1F. The isoelectric point (pI) values of the collagen samples were determined to be 5.40 (ASC) and 5.75 (PSC), respectively. 

Although both ASC and PSC belong to type I collagen and have a complete triple helix structure, there are still some differences in molecule weight, thermal stability and pI value, which may be attributed to the removal of telopeptides. Lower molecular weight and thermal stability suggested that PSC can be more easily degraded and absorbed in vitro. Compared to ASC, the pI of PSC is closer to neutral, which allows it to self-aggregate faster to form hydrogels within a neutral pH range [47,48]. In addition, Kreger et al. [49] have found that ASC polymerized faster than PSC, and noted that telomeres that were damaged or destroyed by pepsin digestion played a key role in fiber nucleation. However, studies have shown that telocollagen (PSC) has lower immunogenicity than the collagen with complete telopeptides (ASC) [50,51,52] and is still able to self-assemble into hydrogels [53]. Therefore, PSC was selected to prepare collagen hydrogel for the evaluation of its mechanical properties and biomedical potential as a wound dressing in the following studies.

### 2.2. Water Content and Water Retention Ratio of Collagen Hydrogels

**Figure 2:**
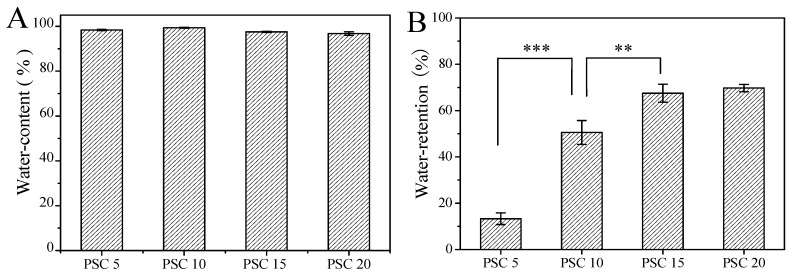
Water content and water retention ratio of collagen hydrogels with different PSC contents. (**A**): Water content; (**B**): Water retention ratio. PSC 5: collagen hydrogels with 5 mg/mL PSC; PSC 10: collagen hydrogels with 10 mg/mL PSC; PSC 15: collagen hydrogels with 15 mg/mL PSC; PSC 20: collagen hydrogels with 20 mg/mL PSC. The ** means *p* < 0.01, and *** means *p* < 0.001.

The ability of a wound dressing to preserve water is an important index to evaluate its property for tissue engineering [54]. According to the “wet wound healing theory”, the ideal wound dressing should keep the wound environment moist [55]. As shown in Figure 2A, all the prepared collagen hydrogels with different PSC content had high water content and there was no significant difference between each group (*p* > 0.05). But the water-retention ratio increased as the PSC content increased from 5 to 20 mg/mL (Figure 2B). These probably occurred because the mesh structures increased in the collagen hydrogels with increased PSC content, and water was locked in the sturdy structure and could not get out of meshes with smaller sizes even under a certain centrifugal force, which was consistent with the following results of rheology.

### 2.3. Rheological Characterization of Collagen Hydrogels

**Figure 3:**
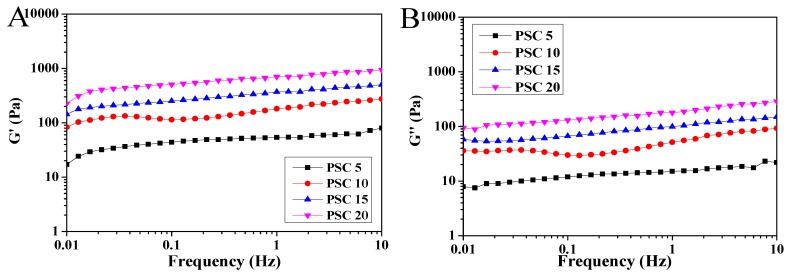
Rheological curves of collagen hydrogels with different PSC contents. (**A**): Storage modulus (G’); (**B**): Loss modulus (G’’); PSC 5: collagen hydrogels with 5 mg/mL PSC; PSC 10: collagen hydrogels with 10 mg/mL PSC; PSC 15: collagen hydrogels with 15 mg/mL PSC; PSC 20: collagen hydrogels with 20 mg/mL PSC.

Rheological curves of collagen hydrogels with different PSC contents were obtained and shown in Figure 3. All experiments were conducted in linear viscoelastic regions. The G’ modulus describes the elasticity, and the G’’ modulus reflects the dissipated energy as a characteristic viscosity [56]. It can be seen that the G’ moduli for each sample (Figure 3A) were larger than the corresponding G’’ moduli (Figure 3B) in the measured frequency range of 1.0–10 Hz, which indicated that each hydrogel showed more elasticity than viscosity. This result was consistent with hydrogels derived from bovine collagen [57] and tilapia fish-scale collagen [56]. In addition, as the PSC concentration increased, the G’ and G’’ of the collagen hydrogels increased significantly, indicating that the mechanical properties of the hydrogels were significantly improved. The values of G’/G’’ were calculated as approximately the same, which may suggest the hydrogels with different PSC content had a similar structure and only show an increase in density [56]. The hydrogels with PSC content of 20 mg/mL showed the best mechanical properties, which could be attributed to the increased microstructure of collagen fibrils.

### 2.4. Scanning Electron Microscopy (SEM) of Collagen Hydrogels

**Figure 4:**
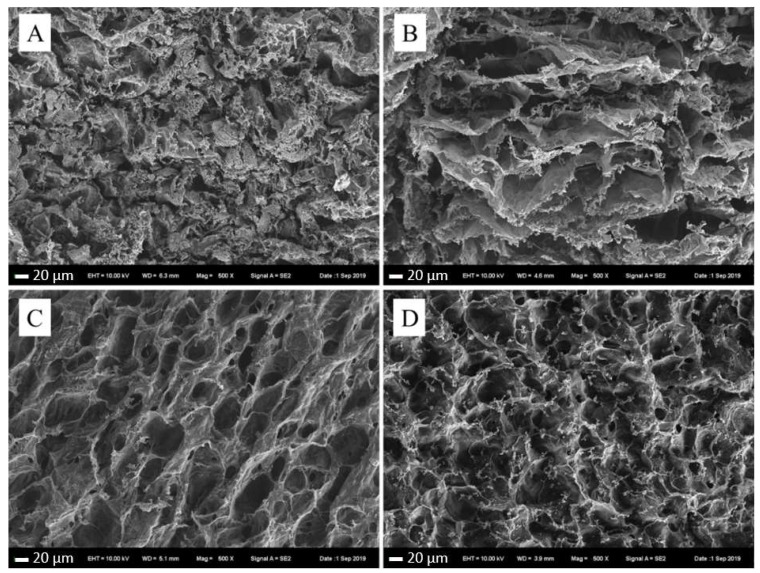
Scanning Electron Microscopy (SEM) images of collagen hydrogels with different PSC contents. (**A**): Collagen hydrogels with 5 mg/mL PSC; (**B**): Collagen hydrogels with 10 mg/mL PSC; (**C**): collagen hydrogels with 15 mg/mL PSC; (**D**): collagen hydrogels with 20 mg/mL PSC. Original magnification: ×500.

The inner pore morphology and connectivity between pores of collagen hydrogel is important for their application for cell seeding, migration, mass transport, cell growth, gene expression and new tissue formation in three dimensions [57]. As shown in Figure 4, the interior morphology of collagen hydrogels exhibited sparse membrane-like mesh structure, and mesh structure got tighter and more regular with the increase of PSC concentration. The internal structure of collagen hydrogel with 5 mg/mL PSC was not as regular as others, which was because of the low mechanical strength and the collapse of the internal structure. Hydrogels with suitable mechanical strength and a porous mesh structure ensure a good permeability, which is beneficial for promoting cell proliferation and wound healing, therefore, the collagen hydrogels with PSC content of 10 mg/mL, 15 mg/mL, and 20 mg/mL may be a potential wound dressing due to their dense and regular network structures.

### 2.5. Cytotoxicity of Collagen Hydrogels

**Figure 5:**
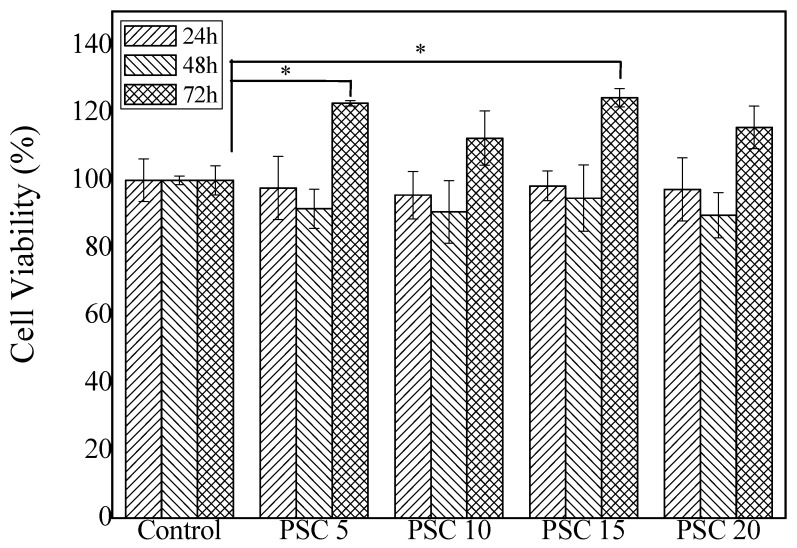
Cytotoxicity evaluation of collagen hydrogels with different PSC contents. PSC 5: collagen hydrogels with 5 mg/mL PSC; PSC 10: collagen hydrogels with 10 mg/mL PSC; PSC 15: collagen hydrogels with 15 mg/mL PSC; PSC 20: collagen hydrogels with 20 mg/mL PSC. * *p* < 0.05.

Fibroblasts play an important role in wound repairing. Various studies have shown that fibroblasts participate in the formation of granulation tissue during the wound healing, synthesizing and secreting of extracellular matrixes, such as fibronectin, hyaluronic acid and collagen [58,59,60]. The biocompatibility and the cytotoxicity of the collagen hydrogels were determined using NIH-3T3 fibroblasts as tested cells. As shown in Figure 5, compared with the blank control group, the cell viability of the hydrogels group with different collagen content decreased slightly (*p* > 0.05) for the first two days. However, after three days of continuous culture, the cell survival rate of the experimental group exceeded 100% (the groups of PSC 5 and PSC 15 showed a significant increase, *p* < 0.05). These suggest that collagen extract from the prepared collagen hydrogels with different PSC concentrations showed no significant cytotoxicity but could also promote the proliferation of fibroblast NIH-3T3, which may be caused by the degradation of collagen. This is consistent with the reports from Aiah [34], where collagen at a concentration of 0.3% and 0.5% (*m*/*v*) was proved to be biocompatible and non-toxic to the active proliferation of BHK-21 cells.

### 2.6. Healing of Deep Second-Degree Burn of Rat Skin

**Figure 6:**
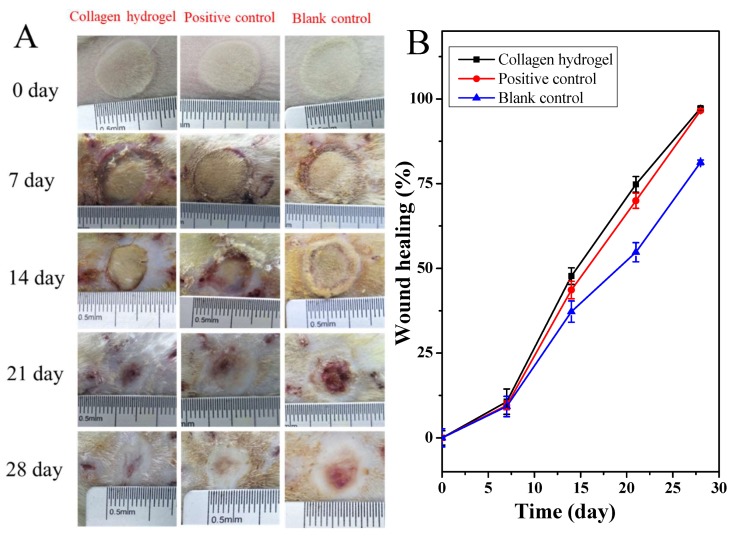
Healing of deep second-degree burn of rat skin with different treatments. (**A**): Photographs of deep second burn wounds at 0, 7, 14, 21 and 28 days. (**B**): Wound healing rate with different treatments. Positive control group, treated with commercial product (3MTM Tegaderm^TM^ hydrocolloid dressing); Collagen hydrogels group, treated with collagen hydrogel dressing containing 10 mg/mL PSC; Blank control group, without any treatment after wound burned.

When the PSC content over 10 mg/mL, the solubility of collagen will be poor, and the collagen solution become very viscous, which makes the preparation of hydrogels difficult. Therefore, the 10 mg/mL PSC hydrogels with a regular network structure, suitable mechanical strength and water retention ratio were used as a wound dressing to evaluate their effect on the healing of deep second-degree burns on rat skin. Figure 6A shows the healing effects of representative animals in each group at 0, 7, 14, 21 and 28 days after scald. Among all these groups, the wound area treated by collagen hydrogels was smaller than other samples from the seventh day after scald. Figure 6B depicts the wound healing rates treated with different methods. The healing rate of the collagen hydrogels group was significantly higher than the blank control groups on the 14th, 21st, and 28th day (*p* < 0.05, *p* < 0.01, *p* < 0.0001, respectively), which is slightly better than the positive control (*p* > 0.05), exhibiting the highest wound healing rate. Christophe et al. [61] have reported that hydrogels with higher collagen content are stable, and can enhance cell viability and allow the gene expression of matrix macromolecules and cytokines involved in neovascularization or re-epithelialization, demonstrating that concentrated collagen hydrogels could be a new candidate for cell therapy in chronic skin wounds. In our study, collagen hydrogels with a PSC content of 10 mg/mL, had a proper mechanical strength and could lock more moisture, and then keep the wound surface in a humid environment, which fulfilled better the requirement of wet wound healing. Our results suggested that the collagen hydrogels from tilapia skins could be used as wound dressings for the treatment of deep second-degree burns.

### 2.7. Histological Analysis

**Figure 7:**
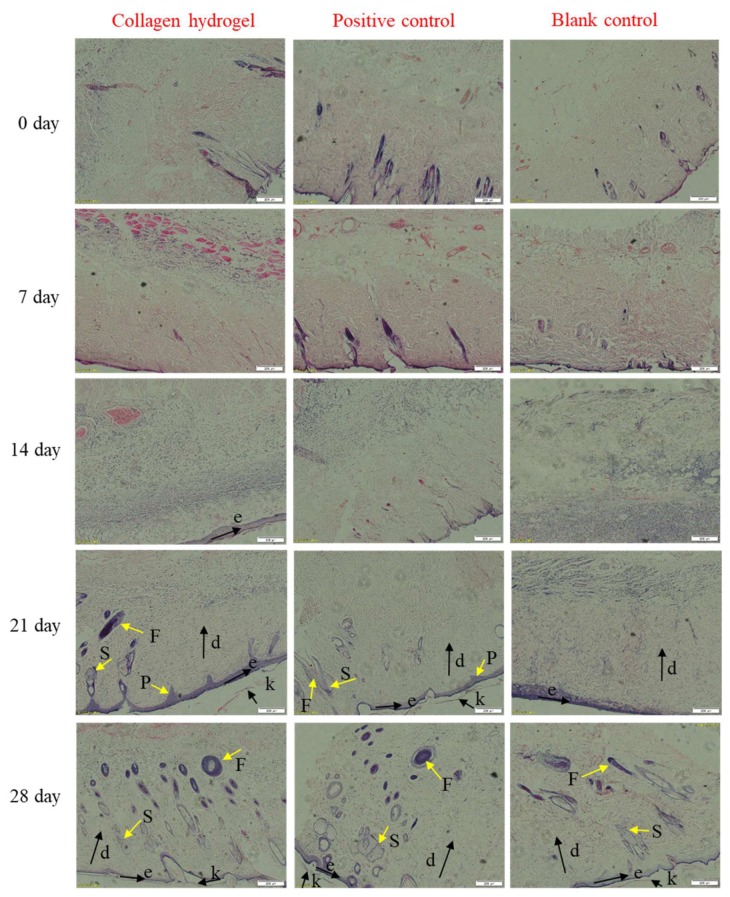
H and E-stained histologic sections of burn wounds at various times. Blank control: without treatment; Positive control: treated with commercial product (3MTM Tegaderm^TM^ hydrocolloid dressing); Collagen hydrogel: treated with 10 mg/mL PSC hydrogels. Abbreviations: P: dermal papillae; F: follicle; S: sebaceous gland; k: keratinized layer; e: epidermis layer; d: dermis. Original magnification: ×100.

**Figure 8:**
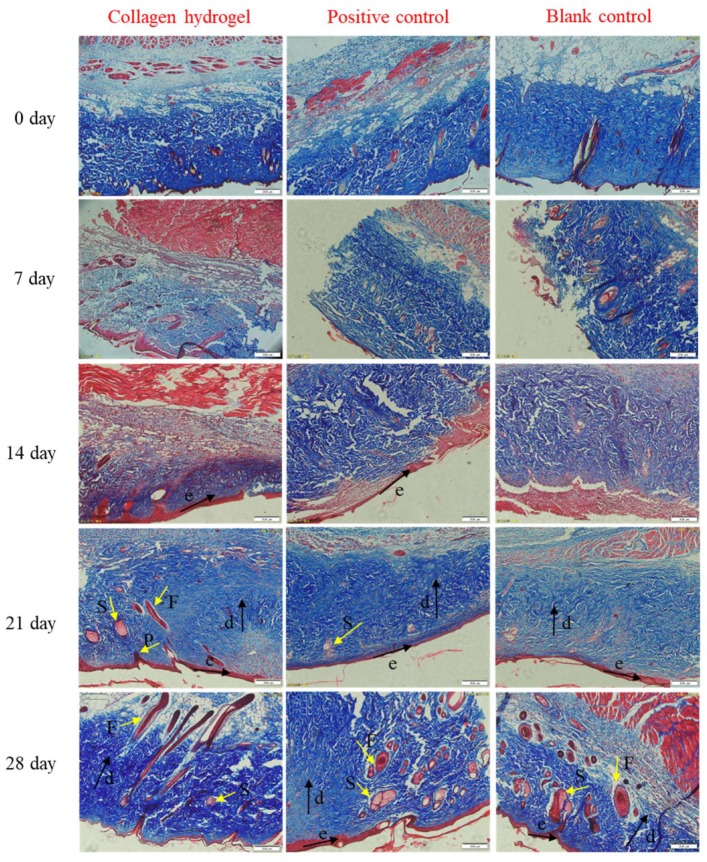
Masson’s trichrome stained histologic sections of burn wounds at various times intervals within 4 weeks. Blank control: without treatment; Positive control: treated with commercial product (3MTM Tegaderm^TM^ hydrocolloid dressing); Collagen hydrogel: treated with 10 mg/mL PSC hydrogels. Abbreviations: P, dermal papillae; F, follicle; S, sebaceous gland; k, keratinized layer; e, epidermis layer; d, dermis. Original magnification: ×100.

Histological analysis was performed by H and E and Masson’s trichrome staining to further evaluate the process of burn healing (Figure 7 and Figure 8). As shown in Figure 7, on day zero (skin tissue taken about 12 hours after burned), the dermal-epidermal junction disappeared and skin appendages were basically damaged, suggesting the successful generation of deep second-degree burn wounds [62]. In the early stage (on day 7) of wound healing, fibrous tissue was all disordered in the three groups. However, on the 14th day the appearance of the new epidermis was firstly seen in the collagen hydrogel group, while no new epidermis was formed in the positive control group and the fibrous tissue in the blank control group was still disordered. The hair follicles, sebaceous glands and dermal papillae could be clearly observed in the collagen hydrogels and commercial product treatment groups within 21 days and it was clearly seen that the skin appendages of the collagen group were more mature, but the blank control group only had epidermis formation and no skin appendages. On the 28th day, ordered fibrous tissue and a large number of skin appendages were observed in the collagen hydrogels and positive control treated groups, but there are a few skin appendages in the blank control group. The faster formation of the epidermis layer and higher maturity of skin accessories suggest that collagen hydrogels had a better effect on promoting burn wound healing than the blank group or commercial product, which may be due to the promotion of collagen on the proliferation and differentiation of epithelial cells. 

By Masson staining, collagens can be stained blue and keratins can be stained red [63]. From Figure 8, it can be seen that the blue area changed from loose and disordered (day 7) to dense and ordered (day 21), which indicates that the collagen was broken and damaged due to burns at the beginning, and the new collagen was rearranged during the wound healing process. These can also be clearly observed from the skin appendages formed in the dermal layer. Compared with the other two groups, the collagen hydrogel treated group formed new collagen and skin appendages in the shortest time, which suggests that the collagen hydrogels could promote skin regeneration and then accelerate the wound healing.

## 3. Materials and Methods 

### 3.1. Materials

Live tilapia fish (*Oreochromis niloticus*) was purchased from the fish market (Yantai, Shandong, China). The skins were mechanically separated in an ice-water mixture, and the residue of the adhering tissues was manually removed. After washed with cold distilled water, and the skins were stored at −20 °C. All reagents used for extraction and isolation were of analytic grade.

### 3.2. Extraction and Characterization of ASC and PSC

The ASC was extracted from tilapia fish skin as previously reported [29]. All processes were carried out at 4 °C with continuous stirring (MS 4, IKA^®^ C-MAG, Staufen, Germany). The extraction of PSC was similar to that of ASC except that the tilapia fish skins were treated by 0.5 M acetic acid containing 0.5% (*w*/*v*) pepsin, and then dialyzed against 0.02 M Na_2_HPO_4_ until the pH value was over 8.0 to inactivate pepsin, and finally dialyzed against 0.1 M acetic acid. The extracted ASC and PSC samples were lyophilized (1-2 LD plus, ALPHR, Freising, Germany) and stored at −20 °C until required. Samples were sterilized with 20 kGy ^60^Cobalt irradiation from Qingdao Irradiation Center and used for biological evaluations, such as cytotoxicity characterization and animal experiments.

The extracted ASC and PSC were characterized using SDS-PAGE [29] (NuPAGETM 4–12% Bis-Tris Gel (Thermo Fisher Scientific, Inc., Waltham, MA, USA)) and FTIR [41] (Nicolet 6700, Thermo Fisher Scientific, USA) to determine the type of collagen and verify the integrity of its triple helix. Molecular conformation of collagen was assessed by CD using a MOS-450 instrument (Bio-Logic, Inc., Cologne, France) according to the method of Sun et al. [64]. UV-Vis [65] (UV-2450, Shimadzu, Kyoto, Japan), differential scanning calorimetry (DSC 200 F3, Netzsch-Gerätebau GmbH, Selb, Germany) [46,66] and zeta potential analysis (Nano-ZS, Malvern, Inc., Malvern, UK) methods [28] were also performed to characterize the absorbance, thermal stability and zeta potential of collagen as previously reported. The weight of lyophilized collagen was about 5–10 mg with an empty aluminum crucible as a reference for DSC.

### 3.3. Preparation and Characterization of Collagen Hydrogel

For collagen hydrogel preparation, the PSC was firstly dissolved with 0.1 M acetic acid at 4 °C. Then 5× phosphate buffered saline (PBS) was slowly added with a collagen solution/PBS ratio of 4:1 (*v/v*) and then mixed thoroughly. After being adjusted to neutral (7.4 ± 0.2), the prepared solution was immediately poured into the molds. The hydrogels with final PSC content of 5 mg/mL, 10 mg/mL, 15 mg/mL and 20 mg/mL, respectively, were obtained by self-aggregation of the collagen at 30 °C for 24 h.

The hydrogels before and after freeze-drying were accurately weighed and marked as *M*_1_ and *M*_2_, respectively. The water content was calculated according to the following formula [67]:

$$\mathrm{Water}\;\mathrm{content}\;\left(\%\right)=\frac{M_{1}{\;-\;M}_{2}}{M_{1}}\;\times \;100\%$$

The hydrogels before and after centrifugation at 5000 g for 3 min was accurately weighed and marked as *M*_3_ and *M*_4_. Hydrogel weight after lyophilization was recorded as *M*_5_. The water retention ratio was calculated according to the following equation [68]:

$$\mathrm{Water}\;\mathrm{retention}\;\left(\%\right)=\frac{M_{4}\;{-\;M}_{5}}{M_{3\;}{-\;M}_{5}}\;\times \;100\%$$

The rheological characterization of collagen hydrogels was performed according to the method of Chen et al. [69] with slight modification. Briefly, 600 μL hydrogel was taken and analyzed using a rheometer (HAAKE MARS, Thermo Scientific, Waltham, MA, USA) with a 35 mm rotor, a 2° taper and corresponding cone plate at 25 °C. The stress scan was first performed to measure the linear viscoelastic regions and then the appropriate stress value was selected for frequency scanning ranging from 1.0 to 100 rad s^−1^. The storage modulus (G’) and the loss modulus (G’’) were automatically recorded.

The morphology of collagen hydrogel was characterized using a scanning electron microscope (SEM 660, FEI Nava Nano, Hillsboro, OR, USA). Samples were prepared and freeze-dried at −50 °C to volatilize any water, and then cut into 1 × 1 cm square pieces and coated with gold. The microstructures of the samples were detected at 3.0 kV.

### 3.4. Cytotoxicity Characterization

The cytotoxicity of collagen hydrogels was carried out as reported by Fiocco et al. [70] with slight modification. Aseptic collagen hydrogels were prepared and lixiviated with L-DMEM (HyClone, Logan, UT, USA) for 72 h at 37 °C with lixiviation ratio as 0.1 % (*w*/*v*). The Cell density of NIH-3T3 fibroblasts was adjusted to 1 × 10^4^ /well, and cultured in a 96-well plate at 37 °C, 5% CO_2_ (HERAcell 150i, Thermo Scientific, USA). After 24 hours of culture, the culture medium of experimental groups was replaced with the lixiviation solution, containing 10% fetal bovine serum (FBS) and 1% penicillin-streptomycin mixture (100 unit/mL penicillin, 100 μg/mL streptomycin) (Solarbio, Beijing, China), and the control group was replaced with L-DMEM containing 10% FBS and 1% penicillin-streptomycin mixture. The percentage of viable cells was detected using the 3-(4,5-dimethyl-2-thiazolyl)-2,5-diphenyl-2-H-tetrazolium bromide (MTT method after 24 h, 48 h, and 72 h of continuous culture. The cell viability was calculated as follows:

$$\mathrm{Cell}\;\mathrm{Viability}\;\left(\%\right)=\frac{A_{t}\;-\;A}{A_{0}\;-\;A}\;\times \;100\%$$

where *A*_0_, *A*_t_ and *A* were the absorbance at 490 nm of control group, experimental group and the group without cells, respectively.

### 3.5. Animal Experiments

In total, 45 adult Sprague-Dawley rats, 250 g ± 20 g in body weight, male and female, were purchased from Jinan Pengyue Laboratory Animal Breeding Co., Ltd. (Approval Number: SCXK 2014 0007). All animal experiments complied with the Institutional Animal Care and Use Committee (IACUC) guidelines. The rats’ skin-deep second-degree burns were prepared according to the method of Li et al. [71]. Before burning, all the rats were firstly raised for 7 d while being fed standard laboratory food and water, and then anesthetized with 0.3 mL of 10% chloral hydrate per 100 g body weight injected through intraperitoneal, and shaved on the back with an electric razor. A small temperature-controlled electric iron with a diameter of 1.3 cm was burned at 100 °C for 10 s on both sides of the rat’s back to generate a deep second-degree scald model. Rats were randomly divided into the drug-administered group (collagen hydrogels), the positive control group (hydrocolloid thin dressing which is widely used in scald clinical applications, 90022T, Tegaderm^TM^, Taiwan, China), and the blank control group, with 15 rats in each group. Each rat was housed in a single cage, and every day the dressing was changed and the wound healing of each group was observed and photographed. Three rats from each group were sacrificed at 0, 7, 14, 21, and 28 days, and the burned area and surrounding skin tissue, about 1.5 cm × 1.5 cm, were taken and fixed in 10% paraformaldehyde for histological examination.

The wound healing of skin scald in rats was photographed and the wound area was analyzed using imageJ 1.52a software. The wound healing was calculated as following equation [72]:

$$\mathrm{Wound}\;\mathrm{healing}\;\left(\%\right)=\frac{A_{0}\;-\;A_{t}}{A_{0}}\;\times \;100\%$$

where *A*_0_ is the original wound area and *A*_t_ is the area of wound at the time of biopsy on days 0, 7, 14, 21 and 28 days, respectively.

### 3.6. Histological Analysis

Burn wound skin tissues were collected, treated with formaldehyde (10%), and embedded in paraffin. Then the samples were cut into sections of 5 μm in thickness by manual rotary slicer (Leica RM 2235, Nussloch, Germany). After being dewaxed and rehydrated, sections were stained with H and E and Masson’s Trichrome Staining kit (Solarbio, Beijing, China), sealed with a neutral resin and then examined using an optical microscope.

### 3.7. Statistical Analysis

The experimental results were presented with mean ± standard deviations, and statistical analysis was performed using IBM SPSS Statistics 25.0 (IBM Co., Armonk, NY, USA) and *p*-values < 0.05 were considered significant.

## 4. Conclusions

Two kinds of collagens, ASC and PSC, were extracted from the tilapia skin and a novel hydrogel wound dressing with 10 mg/mL of PSC was prepared by the self-assembly properties of collagen. The prepared hydrogels contain a regular network structure, suitable mechanical strength, moderate water retention ratio and no obvious cytotoxicity. Animal experiments showed that the collagen hydrogel dressing could significantly accelerate the healing of deep second-degree burn wounds. Compared with commercial products, the collagen hydrogel dressing could promote the formation of epidermal layers and the maturation of skin appendages, which suggests that the collagen hydrogel dressing from the tilapia skin can be developed as a novel and effective wound dressing for the treatment of deep degree burn wounds.

## Figures and Tables

**Table 1:** Peak distribution for FTIR analysis of acid-soluble collagen (ASC) and pepsin-soluble collagen (PSC).

Region	Peak Wavenumber (cm^−1^)		Assignment
	ASC	PSC	
Amide A	3336.25	3343.96	NH stretch coupled with hydrogen bond
Amide B	2942.84	2927.41	CH_2_ symmetrical stretch
Amide I	1660.41	1658.48	C=O stretch/hydrogen bond coupled with COO^−^
Amide II	1552.42	1554.34	NH bend coupled with CN stretch
Amide III	1241.93	1241.93	NH bend coupled with CN stretch
